# Neuropsychiatric symptoms in Posterior Cerebral Artery Stroke: Avoiding misdiagnosis

**DOI:** 10.1192/j.eurpsy.2023.1614

**Published:** 2023-07-19

**Authors:** B. F. Silva, O. Nombora, A. Oliveira

**Affiliations:** Centro Hospitalar Vila Nova de Gaia, Vila Nova de Gaia, Portugal

## Abstract

**Introduction:**

Posterior Cerebral Artery (PCA) strokes cause the restriction of blood flow to multiple areas of the brain including the occipital lobe, the thalamus, the inferomedial temporal lobe, the upper part of the brainstem and the midbrain. This results in a panoply of possible symptomatology (including psychiatric manifestations) that increases the difficulty in diagnosis.

**Objectives:**

We aimed to present and discuss atypical presentations of cerebrovascular disease that often results in misdiagnosis in an emergency context.

**Methods:**

A non-systematic review of the topic was conducted, and a case report is presented.

**Results:**

An 86-year-old male patient, previously autonomous and cognitively intact, presents with periods of confusion and incoherent speech, visual hallucinations, incongruity of affect with pathological laughter, insomnia and increased aggressive behaviour, which began suddenly and worsened in the period of a week. The symptoms motivated several recurrences to the emergency department and numerous diagnostic exams performed, including CT scans and an EEG. Neurological examination showed no focal neurological deficits. The patient was admitted to a psychiatric ward for further diagnostics work-up. Due to increasingly altered status of consciousness, an MRI was performed, which found ischemic left occipital lesions compatible with PCA stroke. The patient was afterwards transferred to a neurology ward for continuing medical care.

**Conclusions:**

This case exemplifies how atypical symptoms such as visual hallucinations and changes in behaviour can be the only clues to diagnosing a PCA infarction, particularly in the absence of other focal neurological deficits. PCA strokes most commonly present with homonymous hemianopia, unilateral limb weakness, gait ataxia and vertigo. However, several other studies and case reports have found that this is not always the case and a minutious approach should be preferred in patients with a sudden onset of sensory and perceptual alterations and oscillating state of consciousness and disorientation, especially when discussing elderly people. Often these patients are admitted in psychiatric wards which may hinder the appropriate care they must receive.

**Disclosure of Interest:**

None Declared

